# MBX-7591: a promising drug candidate against drug-resistant fungal infections

**DOI:** 10.1128/mbio.01361-24

**Published:** 2024-07-11

**Authors:** Nivea Pereira de Sa, Maurizio Del Poeta

**Affiliations:** 1 Department of Microbiology and Immunology, Stony Brook University, Stony Brook, New York, USA; 2 Veterans Administration Medical Center, Northport, New York, USA; 3 Institute of Chemical Biology and Drug Discovery, Stony Brook University, Stony Brook, New York, USA; 4 MicroRid Technologies Inc., Dix Hills, New York, USA; 5 Division of Infectious Diseases, School of Medicine, Stony Brook University, Stony Brook, New York, USA; Duke University, Durham, North Carolina, USA

**Keywords:** *Aspergillus fumigatus*, drug development, fungal infections, fatty acid, membrane

## Abstract

Invasive fungal infections (IFIs) caused by pathogenic fungi pose a significant public health concern, particularly for immunocompromised individuals. Mortality rates for IFIs remain high, and currently available treatment options are limited. Existing antifungal agents often suffer from limited clinical efficacy, poor fungicidal activity within the host, potential toxicity, and increasing ineffectiveness due to emerging resistance, especially against triazole drugs, the current mainstay of antifungal treatment. A recent study has identified MBX-7591, a small molecule with promising antifungal activity against *Aspergillus fumigatus* and other pathogenic fungi, including strains resistant to triazoles (C. Gutierrez-Perez, C. Puerner, J. T. Jones, S. Vellanki, E. M. Vesely, et al., mBio e01166-24, 2024, https://doi.org/10.1128/mbio.01166-24). This novel compound appears to inhibit stearoyl-CoA 9-desaturase, a key enzyme involved in fungal fatty acid biosynthesis. By disrupting the conversion of saturated fatty acids to oleic acid, MBX-7591 offers a unique mechanism of action, potentially reducing the risk of resistance development. Here, we now discuss the implications of these groundbreaking findings for overcoming antifungal drug resistance.

## COMMENTARY

Invasive fungal infections (IFIs) pose a significant threat to immunocompromised individuals, with high mortality rates (1). While significant research has been conducted on the cellular processes underlying fungal pathogenesis, translating this knowledge into effective clinical interventions remains challenging due to the high degree of similarity between fungal and human physiology (2). This similarity often limits the efficacy and safety of existing antifungal agents, which are often poorly fungicidal, exhibit significant toxicity, and are increasingly hampered by emerging resistance (3–5). *Aspergillus fumigatus*, a ubiquitous environmental saprophyte (6), is listed by the World Health Organization as one of the top four critical fungal species requiring urgent development of effective therapies (7). Invasive aspergillosis (IA) affects over 2 million individuals annually, with voriconazole remaining the frontline treatment alongside other triazoles, amphotericin B, and echinocandins (1).

Triazoles are the primary antifungal class used to combat *A. fumigatus*. Their antifungal effect stems from targeting a crucial enzyme in the fungal cell membrane biosynthesis pathway: cytochrome P450 14-α-sterol demethylase (Cyp51A/B) (8, 9). By binding to and inhibiting this enzyme, triazoles prevent the conversion of lanosterol to ergosterol, a vital component of the fungal cell membrane (10). This depletion of ergosterol weakens and disrupts the integrity of the cell membrane, ultimately inhibiting fungal growth (8). Currently, four triazole drugs are employed clinically for the treatment of IA: voriconazole, the most commonly used first-line therapy, followed by itraconazole, posaconazole, and isavuconazole (11). However, a growing concern is the rise in triazole resistance among clinical isolates of *A. fumigatus*, posing a significant challenge to IA therapy (11, 12). The major mechanism of triazole resistance involves mutations in the gene encoding the *A. fumigatus* cytochrome P450 triazole target, *cyp51A* (13). However, recent research has identified mutations affecting the HMG-CoA reductase (HMGCR) enzyme, Hmg1, as another contributing factor, highlighting the complexity of fungal resistance development (12, 14). The development of alternative antifungal strategies is urgent and crucial to address the growing challenge of azole resistance and improve patient outcomes.

In a recent study, Gutierrez-Perez et al. introduced MBX-7591, a novel small molecule with promising potential for antifungal development, specifically targeting the growing challenge of triazole resistance (15). They utilized a cell-based assay to identify molecules that interfere with the sterol-regulatory element binding pathway transcription factor, SrbA. SrbA is required for sterol biosynthesis, hypoxia fitness, iron homeostasis, and azole drug resistance directly regulating the *cyp51A* gene, which encodes 14-α-sterol demethylase, the enzyme targeted by triazoles (16, 17). Resistant fungal strains can increase *cyp51A* expression, leading to triazole resistance. Therefore, inhibiting SrbA could potentially increase susceptibility to triazoles. Notably, the identified compound, MBX-7591, exhibited a synergistic interaction with triazoles, potentiating their activity even against resistant *A. fumigatus* strains.

This study observed that increasing SrbA expression led to decreased susceptibility to MBX-7591, suggesting that its target is regulated by SrbA. Interestingly, MBX-7591 not only promoted an increase in SrbA cellular levels but also interfered with two other transcription factors that co-regulate genes with SrbA: AtrR and HapX. AtrR plays a role in low-oxygen adaptation, virulence, and triazole susceptibility, while HapX negatively regulates *cyp51A* and *srbA* expression (18–20). While MBX-7591 doesn't directly inhibit these genes, its target and/or the molecular response triggered by MBX-7591 appear to be regulated by SrbA, AtrR, and HapX.

Interestingly, a gene ontology analysis combined with data on genes co-regulated by SrbA, AtrR, and HapX revealed that the most significantly affected biosynthetic process was fatty acid synthesis. Gutierrez-Perez et al. then focused their investigation on this pathway to identify the actual target of MBX-7591. As this exciting investigation narrowed down to fatty acids, they measured the levels of palmitic, stearic, and oleic acids. Palmitic acid is converted to stearic acid through Fas1, while stearic acid is converted to oleic acid through stearoyl-CoA 9-desaturase (SdeA). The authors observed depletion of oleic acid levels while palmitic and stearic acid levels increased, suggesting that MBX-7591 likely inhibits SdeA. In addition, the supplementation with oleic acid to the medium rescues the growth in the presence of MBX-7591 reinforcing this might be the protein target.

Later, the fatty acids will culminate in the formation of the different phospholipids in the cell membrane, so it was expected then that the treatment of fungal cells with the compound would be translated into changes in phospholipids composition that would directly affect membrane fluidity. The evidence presented showed an increase in the saturation and a shortening of the palmitic fatty acid in the phospholipids tail composition, and a decrease in oleic acid. Therefore, SdeA is most likely the molecular target of MBX-7591.

The inhibition of SdeA confers a fungicidal effect *in vitro* and potentially *in vivo*, as demonstrated by a reduced fungal burden in the lungs of mice after 24 hours of intranasal conidia challenge in a short-term aspergillosis model. However, the question of whether MBX-7591 remains effective after complete hyphae formation in the lungs in a long-term model remains unanswered. Investigating this question would likely be a key focus in future research to further validate the potential of MBX-7591 as an antifungal candidate in animal models.

Finally, the discovery of MBX-7591 offers a promising avenue for combating the growing challenge of drug-resistant fungal infections. Its unique mechanism of action, targeting SdeA, holds the potential for overcoming the limitations of current antifungal therapies. Further research is crucial to confirm SdeA inhibition by MBX-7591 through enzymatic assays and computational simulations or co-structure determination. The confirmation of the target and understanding of its interactions may help the optimization of MBX-7591 as a drug candidate. Additionally, investigating its efficacy against established fungal infections is essential. However, the promising pre-clinical results, including its fungicidal effect and synergy with existing drugs, suggest its potential as a valuable new antifungal therapy targeting an unexplored target class. Further research and development are needed to fully evaluate its safety and efficacy, but this study represents a significant step forward in the fight against drug-resistant fungal infections.
